# The Asian Cohort for Alzheimer's Disease (ACAD): Clinical Core Protocol for Assessment and Consensus Diagnosis

**DOI:** 10.1002/alz70860_100284

**Published:** 2025-12-23

**Authors:** Pei‐Chuan Ho, Guerry M. Peavy, Victor W Henderson, Yian Gu, Haeok Lee, Walter W. Kukull, Hyun‐Sik Yang, Yun‐Beom Choi, Wai Haung Yu, Dolly Reyes‐Dumeyer, Boon Lead Tee, Clara Li, Jody‐Lynn Lupo, Helena C Chui, Gyungah R Jun, Van Ta Park, Li‐San Wang, Tiffany W Chow, The Asian Cohort for Alzheimer's Disease Study Clinical Core

**Affiliations:** ^1^ University of Pennsylvania Perelman School of Medicine, Philadelphia, PA, USA; ^2^ University of California San Diego, Department of Neurosciences, La Jolla, CA, USA; ^3^ Stanford University, Palo Alto, CA, USA; ^4^ Cognitive Neuroscience Division, Columbia University, New York, NY, USA; ^5^ Meyers College of Nursing, New York University, New York, NY, USA; ^6^ Department of Medicine, University of Washington, Seattle, WA, USA; ^7^ Brigham and Women's Hospital, Harvard Medical School, Boston, MA, USA; ^8^ Englewood Health, Englewood, NJ, USA; ^9^ University of Toronto, Toronto, ON, Canada; ^10^ Department of Neurology, Vagelos College of Physicians and Surgeons, Columbia University, New York, NY, USA; ^11^ University of California, San Francisco, San Francisco, CA, USA; ^12^ Icahn School of Medicine at Mount Sinai, New York, NY, USA; ^13^ University of California San Diego, La Jolla, CA, USA; ^14^ University of Southern California, Los Angeles, CA, USA; ^15^ Boston University Chobanian & Avedisian School of Medicine, Boston, MA, USA; ^16^ University of California San Francisco School of Nursing, San Francisco, CA, USA

## Abstract

**Background:**

The Asian Cohort for Alzheimer's Disease (ACAD) study addresses the underrepresentation of Asian Americans and Asian Canadians (ASACs) in Alzheimer's Disease (AD) research, through its investigation of genetic and non‐genetic AD risk factors in diverse Asian populations, beginning with Chinese, Vietnamese, and Korean communities. Given the dearth of culturally and linguistically appropriate instruments, the ACAD Clinical Core developed a protocol that includes a Data Collection Packet (DCP). The initial protocol focused on virtual procedures to address limitations imposed by the COVID 19 pandemic. Since AD risk factors may differ among ethnic subgroups, the DCP was designed to encourage research participation by Asian older adults and to gain important information about these risk factors in Chinese, Vietnamese, and Korean populations in the US and Canada.

**Method:**

DCP development was led by the ACAD Clinical Core, composed of clinicians and researchers in medicine, nursing, genetics, neurosciences, and neuropsychology. At least one clinician from each ACAD recruitment site participated in protocol development. The Clinical Core worked closely with the Data Management, Training and Quality Assurance and Biosample Core to ensure successful DCP administration, sample collection, and electronic database entry.

**Result:**

The DCP is available in Cantonese, Korean, Mandarin, and Vietnamese and includes questions about demographics, lifestyle factors (e.g., diet, sleep, physical activities), cognitive and functional abilities, psychiatric symptoms, and medical history. This information, combined with detailed data from a neurological examination, enables study teams to reach a consensus diagnosis of Normal Control (with or without subjective cognitive concerns), Mild Cognitive Impairment, or Dementia, based on criteria described by the National Alzheimer's Coordinating Center, with a dementia diagnosis differentiated as to etiology. The DCP is designed to be culturally and linguistically appropriate, generation‐specific, flexible in scheduling, and sensitive to participant anxiety and fatigue to facilitate participation. As of January 2025, ACAD has consented 1,173 participants, collected 885 samples (424 saliva and 461 blood), and completed 864 diagnoses (109 dementia, 161 MCI, 594 control).

**Conclusion:**

To bridge the gap of ASACs in AD research, ACAD developed the DCP and sample collection protocols. The pilot phase showcased successful virtual assessments, with expanded recruitment planned for Spring 2025.

